# DltX of *Bacillus thuringiensis* Is Essential for D-Alanylation of Teichoic Acids and Resistance to Antimicrobial Response in Insects

**DOI:** 10.3389/fmicb.2017.01437

**Published:** 2017-08-03

**Authors:** Rita Kamar, Agnès Réjasse, Isabelle Jéhanno, Zaynoun Attieh, Pascal Courtin, Marie-Pierre Chapot-Chartier, Christina Nielsen-Leroux, Didier Lereclus, Laure el Chamy, Mireille Kallassy, Vincent Sanchis-Borja

**Affiliations:** ^1^INRA, UMR1319 Micalis Jouy-en-Josas, France; ^2^AgroParisTech, UMR Micalis Jouy-en-Josas, France; ^3^Laboratoire de Génétique de la Drosophile et Virulence Microbienne, Université Saint-Joseph Beirut, Lebanon

**Keywords:** *B. thuringiensis*, *dltX*, antimicrobial peptides, virulence, D-alanylation, insects, innate immunity

## Abstract

The *dlt* operon of Gram-positive bacteria is required for the incorporation of D-alanine esters into cell wall-associated teichoic acids (TAs). Addition of D-alanine to TAs reduces the negative charge of the cell envelope thereby preventing cationic antimicrobial peptides (CAMPs) from reaching their target of action on the bacterial surface. In most gram-positive bacteria, this operon consists of five genes *dltXABCD* but the involvement of the first ORF (*dltX*) encoding a small protein of unknown function, has never been investigated. The aim of this study was to establish whether this protein is involved in the D-alanylation process in *Bacillus thuringiensis*. We, therefore constructed an in frame deletion mutant of *dltX*, without affecting the expression of the other genes of the operon. The growth characteristics of the *dltX* mutant and those of the wild type strain were similar under standard *in vitro* conditions. However, disruption of *dltX* drastically impaired the resistance of *B. thuringiensis* to CAMPs and significantly attenuated its virulence in two insect species. Moreover, high-performance liquid chromatography studies showed that the *dltX* mutant was devoid of D-alanine, and electrophoretic mobility measurements indicated that the cells carried a higher negative surface charge. Scanning electron microscopy experiments showed morphological alterations of these mutant bacteria, suggesting that depletion of D-alanine from TAs affects cell wall structure. Our findings suggest that DltX is essential for the incorporation of D-alanyl esters into TAs. Therefore, DltX plays a direct role in the resistance to CAMPs, thus contributing to the survival of *B. thuringiensis* in insects. To our knowledge, this work is the first report examining the involvement of *dltX* in the D-alanylation of TAs.

## Introduction

The cell wall of gram-positive bacteria contains highly charged anionic polymers called teichoic acids (TAs) that consist of alditol phosphate repeats. These polymers are either anchored to the cytoplasmic membrane via a lipid anchor [lipoteichoic acid (LTA)] (Fischer, 1988; Fischer et al., 1990) or covalently linked to *N*-acetylmuramic acid residues of the peptidoglycan layer [wall teichoic acid (WTA)] (Xia et al., 2010; Brown et al., 2013). The best characterized structures of WTA are poly(glycerolphosphate) and poly(ribitolphosphate) found in *Bacillus subtilis* and *Staphylococcus* *aureus* respectively (Fischer and Rösel, 1980; Reichmann and Gründling, 2011). Regarding LTA, the most common structure is poly(glycerolphosphate) (Schneewind and Missiakas, 2014). The function of TAs is not fully understood; they have long been considered to be essential for Gram-positive bacteria. However, this is no longer the case since mutants of *S. aureus or B. subtilis* devoided of WTA have been obtained (D’Elia et al., 2006a,b). Nevertheless, they play crucial roles in cell morphology and division and are important for many cell envelope-dependent processes such as the activity of autolytic enzymes, the binding of divalent cations, and susceptibility to innate host defenses (Weidenmaier and Peschel, 2008). WTAs and LTAs can be covalently modified by substitutions with either glycosyl residues or D-alanyl esters, or both (Fischer and Rösel, 1980; Fischer et al., 1990). The high prevalence of D-alanylation among Gram-positive species suggests that D-alanylation of TAs is biologically relevant, especially for pathogenic bacteria in the context of host–pathogen relationship (Abachin et al., 2002; Chan et al., 2007). Indeed, covalent addition of cationic molecules such as D-alanine allows bacteria to adjust their net negative charge leading to the repulsion of positively charged host immune factors such as cationic antimicrobial peptides (CAMPs) (Peschel, 2002; Nizet, 2006). CAMPs are positively charged antimicrobial molecules that participate in the first line of defense of a wide variety of organisms ranging from prokaryotes to mammals (Nguyen et al., 2011). CAMPs, that also have hydrophobic domains, are electrostatically attracted by the negatively charged surface of the microbial cell envelope, where they get embedded into the hydrophobic regions of the lipid membranes, thereby causing membrane damage and ultimately leading to cell death (Yount and Yeaman, 2013). CAMPs, such as cecropin, defensin, and defensin-like peptides, are present at all sites in the human body that are routinely exposed to microbes (skin, mucosae, neutrophils, eosinophils, and platelets). Consequently, many pathogens have developed resistance strategies involving the reduction of the cell envelope negative charge, thereby decreasing electrostatic interactions between CAMPs and the negatively charged TAs that are key components of their cell envelope (Schneewind and Missiakas, 2014). The incorporation of D-alanine esters into TAs to partially neutralize the negative charge of their cell walls represents one of the most common bacterial resistance mechanisms that depend on such charge modifications (Fischer, 1994; Collins et al., 2002; Poyart et al., 2003; Kovács et al., 2006; Abi Khattar et al., 2009; McBride and Sonenshein, 2011).

The process of D-alanylation is accomplished by the gene products of an operon containing four to five genes, *dlt(X)ABCD*, that is highly conserved among nearly all gram-positive bacteria (Perego et al., 1995; Neuhaus et al., 1996). These genes have also been found in a few Gram-negative bacteria, notably in soft-rot plant pathogenic enterobacteria (*Dickeya* and *Pectobacterium* spp.), where they have been shown to confer resistance to AMPs, probably by modifying the surface lipopolysaccharides (LPSs) (Pandin et al., 2016). DltA is a D-alanine-D-alanyl carrier protein ligase that catalyzes the D-alanylation of the D-alanyl carrier protein DltC (Heaton and Neuhaus, 1992, 1994; Debabov et al., 1996). The roles of DltB and DltD are less clear. Two models have been proposed: according to Neuhaus and Baddiley, DltD is thought to facilitate D-alanine ligation to DltC and DltB is believed to be involved in the translocation of Alanylated-DltC across the membrane where it may then transfer D-alanine directly onto LTA (Debabov et al., 2000; Neuhaus and Baddiley, 2003). A second model has been proposed, by Fisher and colleagues, to account for the contributions of DltB and DltD; in this model DltB transfers D-alanine from DltC to undecaprenyl-phosphate (C_55_-P) to produce D-Ala-P-C_55._ This lipid linked intermediate is, then, flipped across the membrane, whereas DltD, active at the outer side of the membrane, transfers D-Ala to LTA (Fischer, 1994). Reichmann et al. (2013) recently reexamined the function of the *dlt* operon and found that DltC does not pass through the membrane and, therefore, that it is unlikely that this protein is involved in the final D-alanylation step of LTAs. They also showed that DltD is targeted to the outside of the cell as was suggested by Fisher and colleagues in their model. Nevertheless, the existence of a D-Ala-P-C_55_ lipid linked intermediate has never been confirmed experimentally. In *Dickeya dadantii*, the *dltB* mutant is more sensitive to antimicrobial peptides than the wild type (WT) strain, but a *dltD* mutant is as resistant as the WT strain (Pandin et al., 2016).

Mutants with TAs lacking D-alanine esters exhibit a variety of phenotypic changes. For example, mutants of *S. aureus* lacking a functional *dlt* operon are deficient in their ability to regulate the anionic charge of the outer envelope and show poor survival both when they are exposed to CAMPs *in vitro* and when they are introduced to a host *in vivo* (Peschel et al., 1999). Similarly, mutants of the intracellular pathogen *Listeria monocytogenes* lacking functional *dltA* are highly susceptible to CAMPs (Abachin et al., 2002). *B. subtilis* also uses *dlt* to modify TAs, and thus reduce its sensitivity to CAMPs (Perego et al., 1995). In *Bacillus anthracis*, the *dlt* genes are turned on during spore germination in the host and are necessary for resistance to innate host defenses (Fisher et al., 2006). We have also previously shown that a *Bacillus cereus* mutant lacking a functional *dlt* operon is highly sensitive to colistin and polymyxin B, two standard antimicrobial compounds that have long been used to define the mechanisms by which CAMPs kill bacteria. Virulence of this mutant in an insect model was also significantly attenuated (Abi Khattar et al., 2009).

The *dlt* operon has been studied in many organisms and inactivation of any single gene (*dltA* to *dltD*) is enough to completely abrogate operon function (Peschel et al., 1999; Collins et al., 2002; Vélez et al., 2007; Abi Khattar et al., 2009). In *B. subtilis*, each gene of the *dltABCD* operon is required for the D-alanylation of LTA and these four proteins are believed to be the only proteins that are necessary and sufficient for D-Ala incorporation (Perego et al., 1995). However, in *B. subtilis*, as in most gram-positive bacteria, the operon contains a fifth gene encoding a small protein of less than 50 amino acids in length, DltX. Wang et al. (2004) using a mathematical algorithm have predicted that this small ORF upstream from *dltA* is part of the *dlt* operon and several experimental analyses, in several species, have shown that, all five genes of the operon belong to the same transcriptional unit (Koprivnjak et al., 2006; Palumbo et al., 2006; Bron et al., 2012). Nevertheless, despite being encoded upstream of *dltA* in several gram-positive bacteria, the role of DltX in TAs D-alanylation has not yet been investigated. Therefore, the aim of this study was to determine whether *dltX* encodes a novel protein with a role in D-alanylation of TAs. We therefore constructed a Δ*dltX* mutant strain in *Bacillus thuringiensis* strain 407 (*Bt* 407), by allelic replacement, without affecting the expression of the other four genes of the operon. We then analyzed the effect of the deletion on the morphological and physicochemical properties of the bacterial surface, and examined its effects on bacterial virulence and resistance to host immunity. We report here that the *dltX* of *B. thuringiensis* is essential for the incorporation of D-alanyl esters into TAs and is required for resistance to CAMPs and for full virulence of the bacterium following its injection into the model insects *Galleria mellonella* and *Drosophila melanogaster.*

## Materials and Methods

### Bacterial Strains and Growth Conditions

The acrystalliferous strain *B. thuringiensis* 407 Cry- belonging to serotype 1 (Lereclus et al., 1989) was used throughout this study and designated as *Bt* 407. *Escherichia coli* K-12 strain TG1 was used for the construction of plasmids and cloning experiments, and *E. coli* ET 12567 Dam- Dcm- (Stratagene, La Jolla, CA, United States) was used to generate unmethylated DNA for the electrotransformation of *Bt* 407. *E. coli* and *Bt* 407 cells were transformed by electroporation as previously described (Dower et al., 1988; Lereclus et al., 1989). *Bacillus* strains were grown at 30°C in Luria Broth (LB) or in HCT, a sporulation-specific medium (Lecadet et al., 1980). *E. coli* strains were grown at 37°C in LB. For electrotransformation experiments, *Bt* 407 was grown in brain heart infusion broth (Difco). Media for bacterial selection were supplemented with 50 or 100 μg/mL X-Gal (5-bromo-4-chloro-3-indolyl-β-D-galactopyranoside) for *E. coli* and *B. cereus*, respectively, and with 100 μg/mL ampicillin (Amp) for *E. coli*, and 200 μg/mL kanamycin (Km) or 10 μg/mL erythromycin for *B. cereus* as needed.

### Manipulation of DNA

Chromosomal DNA was extracted from *Bacillus* cells harvested in the mid-exponential growth phase, with the GENTRA Puregene DNA Purification bacteria Kit (QIAgen, France). Plasmid DNA was extracted from *E. coli* by a standard alkaline lysis procedure using QIAprep spin columns (QIAgen, France). Restriction enzymes and T4 DNA ligase (New England Biolabs, United States) were used in accordance with the manufacturer’s recommendations. Oligonucleotide primers used in this study are listed in **Table 1** and were synthesized by Sigma-Proligo (Paris, France). PCR was performed in a thermocycler, Applied Biosystems 2720 Thermal cycler (Applied Biosystems, United States). Amplified fragments were purified with the QIAquick PCR purification Kit (QIAgen). Digested DNA fragments were separated on 1% agarose gels and were extracted from gels with the QIAquick gel extraction Kit (QIAgen). Nucleotide sequences were determined by Beckman Coulter Genomics (Takeley, United Kingdom). Electroporation to transform *Bt* 407 was carried out as previously described (Lereclus et al., 1989).

**Table 1:** Primer sequences used in this study.

Oligo name	Nucleotide sequence (5′–3′)
dltX-a	CATGCCATGGTCGCAATTACTTTCCCTTGC
dltX-b	CAATTTGTTCTAATAACTTCATGTCTTTCCCTCTCTTAATG
dltX-c	CATTAAGAGAGGGAAAGACATGAAGTTATTAGAACAAATTG
dltX-d	GAAGATCTACGCTTGGACCGACAATTAC
Comp-F	GCTCTAGAAATGCCTTCTCCATTTCACG
Comp-R	CCCCCCGGGGTCTTTGCGGACTAGTTTGG
XbaI-dlt-gfp	CGTCTAGAACCCATTGTGTGAGTGGTCG
EcoRI-dlt-gfp	CGGAATTCACCCATTACAAATTCATGCTG
BCF1372.Q3	AATTGAAAAGTGGGCTGCAGAA
BCR1372.Q3	CGCATCTCGCCAAACAAAA

*^∗^Restriction sites are underlined.*

### Construction of a *dltX* Deletion Mutant

A *B. thuringiensis* strain containing a *dltX* deletion was generated by precise, in frame allelic exchange and deletion replacement without antibiotic resistance cassettes. The thermosensitive plasmid MAD (pMAD) was used in these experiments. The 846 bp sequence immediately upstream from *dltX* was amplified with the primers dltX-a, dltX-b, and a 1064 bp sequence immediately downstream from dltX was amplified with the primers dltX-c and dltX-d. The primers dltX-b and dltX-c introduce overlapping PCR products. The two amplicons were then subjected to another PCR cycle with the primers dlt-a and dlt-d., such that a modified *dlt* operon, from which 159 bp had been deleted, was amplified. This amplicon was digested with NcoI and BglII and was introduced between the corresponding cloning sites of pMAD. *Bt* 407 was then transformed with 10 μg of the recombinant plasmid by electroporation as previously described (Lereclus et al., 1989). Transformants were subjected to allelic exchange by homologous recombination and bacteria sensitive to erythromycin, resulting from double crossing over event in which the chromosomal *dltX* copy was replaced with the overlapping sequences, were selected. The procedure for selection of mutants by allelic exchange via double crossover has been described previously (Bravo et al., 1996). Chromosomal allelic replacement in the *dltX* mutant was confirmed by DNA sequencing of the PCR fragments generated from the primer pairs dltX-a and dltX-d, and the resulting *dltX*-deficient strain was designated 407Δ*dltX*.

### Complementation of the *dltX* Mutant

For the complemented strain, the entire *dltX* open reading frame of *Bt* 407 strain with its promotor region was amplified from genomic DNA of *Bt* 407 by PCR with Taq DNA polymerase (Extensor High-Fidelity; Thermo Scientific). The forward primer Comp F included a restriction site for XbaI and the reverse primer Comp R included a restriction site for XmaI. The resulting 611 kb fragment was digested with XbaI and XmaI, gel-purified, and ligated to the pHT304-18 shuttle vector previously digested with the same enzymes. An aliquot of ligation mixture (∼50 ng DNA) was used to transform *E. coli* TG1 by electroporation. The resulting construct, pHT304-18Ω*dltX*, was verified by restriction mapping and transferred into *E. coli* ET 12567 by electroporation. Unmethylated plasmid pHT304-18Ω*dltX* from *E. coli* ET 12567 was then introduced into strain *Bt* 407*ΔdltX* by electroporation.

### Construction of *Pdlt*Ω*gfp* Fusion Reporter Plasmid

The upstream promoter region of the *dlt* operon (Pdlt) was amplified by PCR with Taq DNA polymerase (New England Biolabs) and with the primer pair XbaI-dlt-gfp and EcoRI-dlt-gfp (**Table 1**). The 239 bp PCR product was digested with XbaI and EcoRI, and ligated into the same sites upstream of the promoterless *gfp* gene carried on pHT315Ωgfp (Daou et al., 2009) resulting in the creation of the plasmid pHT315ΩPdlt-gfp. The plasmid was verified by restriction analysis, PCR, and sequencing and was transferred into *E. coli* ET 12567 by electroporation. Unmethylated plasmids from *E. coli* ET 12567 were then introduced into *Bt* 407 and *Bt* 407*ΔdltX* mutant strains by electroporation.

### Quantitative RT-qPCR

The amounts of *dltA* transcripts in Bt407 and *ΔdltX* strains were measured by real time reverse transcription. RNA extraction and cDNA synthesis were performed as described previously (Réjasse et al., 2012). Primers BCF1372.Q3 and BCR1372.Q3 located inside the *dltA* gene were designed with Primer Express software from Applied Biosystems. Real time PCR was carried out with Sybr green PCR master mix (Applied Biosystems) as recommended by the supplier. Mean values were calculated from two separate experiments in which qPCR reactions were performed in triplicate. The cycle threshold was used to determine the relative *dltA* gene expression levels in the two genetic backgrounds. Data were analyzed by the comparative threshold cycle (ΔΔCt) method with the Relative Expression Software Tool (REST 2009 V2.0.13, QIAgen). Expression ratios were normalized to two *Bt* 407 endogenous reference housekeeping genes, *pur* and *tpi*.

### Scanning Electron Microscopy (SEM) Analysis

Scanning electron microscopy (SEM) was performed at the Microscopy and Imaging Platform MIMA2 (Micalis, B2HM, Massy, France) of the INRA research center of Jouy-en-Josas (France). Samples were fixed with 3% glutaraldehyde in 0.1 M sodium cacodylate buffer at pH 7.4 at room temperature (RT) for 1 h. They were then washed three times for 5 min with a solution of 0.1 M sodium cacodylate. Samples were progressively dehydrated with increasing concentrations of ethanol (50%–70%–90%–2 × 100%) at RT for 10 min in each bath, except for 70% (35 min). Samples were critical point dried at 75 bar and 37°C in a Quorum Technologies K850 device (Elexience, France). Liquid CO_2_ was used as the transition fluid. Coupons were mounted on aluminum stubs with double-sided sticky tape. Samples were sputter coated in Ar with Pt (30 nm of thickness) in a Polaron SC7640 device (Elexience, France) at 10 mA and 0.8 kV for 200 s. Observations were performed on a FE-SEM S4500 (Hitachi, Japan) with a sample holder tilted at 45° and a low SE detector, at 2 kV and 16 mm WD.

### D-alanine Quantification of TAs by High-Performance Liquid Chromatography

Strains were grown in LB medium to an OD of 2. A volume of 100 mL of cells was pelleted by centrifugation, washed twice with ammonium acetate buffer (20 mM; pH 4.7), and resuspended in 5 ml of the same buffer. Cells were heat-inactivated (10 min, 100°C) and lyophilized. D-alanine was released from whole cells by mild alkaline hydrolysis, as reported previously (Kristian et al., 2005). Mild alkaline hydrolysis was carried out with 10 mg of dried cells at 37°C for 1 h in 150 μL NaOH at 0.1 N. After neutralization with 0.1 N HCl, the cells were removed by centrifugation and the supernatant was dried under vacuum, and used for precolumn derivatization with Marfey’s reagent (1-fluoro-2,4-dinitrophenyl-5-L-alanine amide; Sigma) as described previously (Kochhar and Christen, 1989). Marfey’s reagent reacts with the optical isomers of amino acids to form diastereomeric *N*-aryl derivatives that can be separated by high-performance liquid chromatography (HPLC). Amino acid derivatives were separated on a C18 reversed-phase column (Hypersil 100, 250 mm × 4.6 mm, 5 μm, Thermo) at 30°C with a Waters HPLC system. Elution was performed with a linear gradient of acetonitrile in sodium acetate buffer (20 mM, pH 4) as described previously (Kristian et al., 2005). Absorbance of the eluate was monitored at 340 nm. D-Ala derivatives were identified by their retention time and quantified using an external standard calibration curve.

### Electrophoretic Mobility

Bacteria were suspended in 1.5 × 10^-3^ M sodium chloride at a concentration of 10^8^ CFU to measure electrophoretic mobility. The pH of the suspension was adjusted to pH 7 with nitric acid (HNO_3_). Electrophoretic mobility was measured with a 50-V electric field and a Laser Zetameter (Zetaphoremetre II; Societé d’Etude Physico-Chimiques, Limours, France). The results were assessed from an automated video of about 200 particles for each measurement. Each experiment was performed in duplicate with two independently prepared cultures. The typical standard deviation for the electrophoretic mobility mean was 0.25 × 10^-8^ m^2^s^-1^V^-1^.

### *In Vivo* Pathogenicity Assays

#### Virulence in Galleria mellonella

Pathogenicity assays were carried out with bacterial vegetative cells (OD 600 = 1) grown in LB liquid medium and *G. mellonella. G. mellonella* eggs were hatched at 25°C and the larvae were reared on beeswax and pollen (La Ruche Roannaise, Roanne, France). For the infection experiments, the larvae are starved 24 h prior to infection and 10^4^ bacterial vegetative cells suspended in 10 μl PBS buffer were injected into the hemocoel of last instar *G. mellonella larvae* (weighing about 200 mg) with a 0.5-by-25 mm needle and a microinjector (KdScientific syringe pump). The larvae in the control group were injected with PBS buffer. Following inoculation, the larvae were kept by groups of five in small Petri-dishes without food (Bouillaut et al., 2005) and mortality was recorded after 48 h of incubation at 30°C. For each strain, 20 larvae were used for toxicity assay and the results shown are the means of at least three independent experiments.

#### Virulence in *Drosophila melanogaster*

The WT Oregon R strain and the *relish^E20^* mutant strain (impaired in IMD signaling and production of antimicrobial peptides) were used in this analysis (Hedengren et al., 1999). Overnight bacterial cultures were washed in PBS and diluted to OD 600 = 2. Twenty adult female flies aged between 2 and 5 days were pricked with a thin tungsten needle formerly dipped into the bacterial preparation as previously described (Ligoxygakis et al., 2002). Surviving flies were counted every 2 h.

### Minimal Inhibitory Concentrations

Polymyxin B has long been used to define the mechanisms by which AMPs kill bacteria [46]. Susceptibility to polymyxin B was evaluated by determining the half inhibitory concentration (IC_50_) from dose-response curves obtained with various concentrations of polymyxin B. The tests were performed in 96-well microplates containing 7–9 concentrations (from 200 to 800 μg/ml) of polymyxin B (Sigma) for WT 407 and 407*ΔdltX* complemented strains, and from 3 to 25 μg/ml for 407*ΔdltX*. Bacterial growth was scored after inoculation of strains at an initial OD_600_ of 0.1 and incubation at 30°C for 6 h. IC_50_ corresponds to the concentration of polymyxin B required to inhibit inoculum viability by half and was determined as the concentration required to bring the curve down to point half way between its top and bottom plateau. The results shown are the means of at least three independent experiments performed in duplicate.

### Statistical Analysis

Results concerning D-alanine quantification of TAs, electrophoretic mobility and *in vivo* pathogenicity assays were analyzed by the two-tailed Student’s *t*-test. A *p*-value of 0.05 was considered to be significant.

## Results

### *In Silico* Analysis of DltX

**FIGURE 1:**
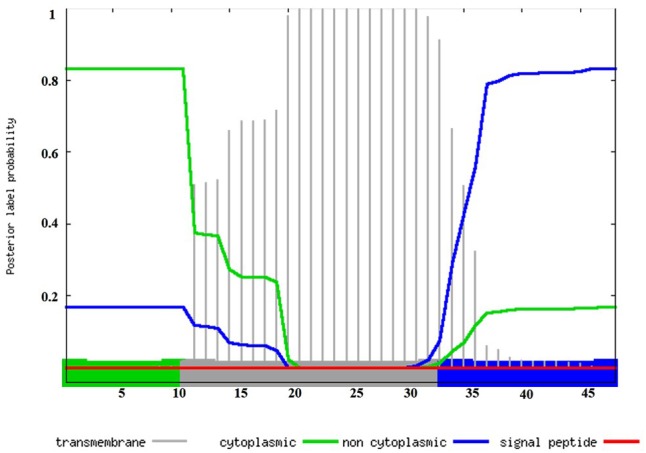
*In silico* analysis of DltX by Phobius, a combined transmembrane protein topology and signal peptide predictor. Transmembrane, cytoplasmic, and non-cytoplasmic domains are represented in the chart. The plot shows the posterior probabilities of cytoplasmic/non cytoplasmic/TM, helix/signal peptide. Predicted transmembrane (amino acids 12 to 33: LTQWVAKTVYYLAILFALLWLY) regions are shown in gray, cytoplasmic and non-cytoplasmic regions are shown in green and blue respectively (amino acids 1 to 11: MERLKEIWSRP and amino acids 34 to 48: GFHDTNTSTFIYNEF). The prediction gives the most probable location and orientation of transmembrane helices in the sequence.

*In silico* BLAST searches indicate that the *dltX* gene is present in 809 species of which 805 are Firmicutes, including *Bacillus, Staphylococcus, Listeria, Lactobacillus, Streptococcus*, and *Enterococcus.* In most cases, *dltX* is located immediately upstream from *dltA*. In *B. thuringiensis* this operon consists of five genes *dltXABCD.* In strain Bt 407, transcripts of *dltX* have been detected by mapping RNA-seq datasets, obtained *in vitro* 2 and 5 h after the end of exponential phase and *in vivo* in infected *G. mellonella* cadavers, 36 h *post mortem*, with the *Bt* 407 reference genome (Sébastien Gélis-Jeanvoine, personal communication). These data prompted us to carry out a detailed analysis of the role of this protein in the process of D-alanylation. DltX belongs to the DUF3687 superfamily of proteins. To date, 811 sequences with this domain are listed in Pfam. Proteins in this family are approximately 50 amino acids in length and their protein sequences are highly conserved among the firmicutes. There are two completely conserved residues (L and Y, at positions 23 and 45 of the protein, respectively) that may be functionally important. A number of entries are annotated as D-Ala-teichoic acid biosynthesis protein; however, there is no direct evidence to support this annotation. In *Bt* 407, DltX contains 11 positively charged amino groups clustered near the N-terminus followed by a hydrophobic region of 22 amino acids and by a putative non-cytoplasmic domain of 15 amino acids in the C-terminus. The 22 amino acids hydrophobic region is predicted to form an α-helix, which suggests that DltX is a transmembrane protein. The topology of DltX present in the Gram negative species is the same, although there is no sequence similarity. In addition, DltX does not have a predicted signal peptide, which suggests that it is probably not exported (**Figure 1**).

### Deletion of *dltX* Does Not Affect the Expression of the *dlt* Operon

**FIGURE 2:**
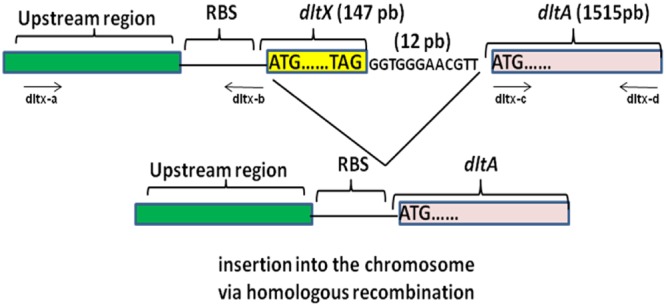
Schematic diagram showing the construction of an in frame deletion of the *dltX* gene by Splicing by Overlap Extension (SOE). The top part of the diagram shows the DNA fragment containing the upstream and downstream DNA regions flanking *dltX*. Synthetic oligonucleotides are represented by lines with arrows indicating the 5′–3′ orientation. Oligo dltX-b 3′ and oligo dltX-c 5′ match their respective template DNA sequences in their 3′ portions and are complementary to each other in their 5′ portions. The double stranded DNA products generated in separate PCR reactions were denatured, allowed to anneal at their overlap and were 3′ extended by DNA polymerase. The fusion product was then further amplified by PCR in the presence of oligo dltX-a and oligo dltX-d.

We sought to study the influence of *dltX* on teichoic acid D-alanylation; therefore, we constructed a mutant harboring a precise in frame allelic deletion of the *B. thuringiensis dltX* gene, to avoid impact on the transcription of the downstream located *dlt* genes. We used the overlap extension (OE-PCR) method, which introduces a deletion without the use of antibiotic resistance cassettes (**Figure 2**). As a result of this deletion, the other genes of the operon are placed directly under the control of the upstream promoter region and are not affected in transcription. We first investigated WT and Δ*dltX* mutant growth dynamics by following optical density (at 600 nm) in liquid cultures and did not detect any significant differences in bacterial growth and cell doubling time. We then used a reporter construct in which the *dlt* upstream promoter region was fused to *gfp*, to investigate whether the deletion of *dltX* affects the transcriptional regulation of the *dlt* operon. We introduced the construct into WT and Δ*dltX* mutant backgrounds and quantified *gfp* expression in both strains. We found no differences in the expression of *Pdlt*Ω*gfp* fusions (data not shown) between strains. We also performed RT-qPCR to examine the abundance of *dltA* mRNA, in WT and Δ*dltX* strains, during the mid-exponential growth phase in standard LB medium. We observed no differences in *dltA* expression between WT and *dltX* mutant cells. Together, these findings indicate that DltX does not affect the transcription of the operon.

### Morphological Characteristics of WT and the *dltX* Deletion Mutant

**FIGURE 3:**
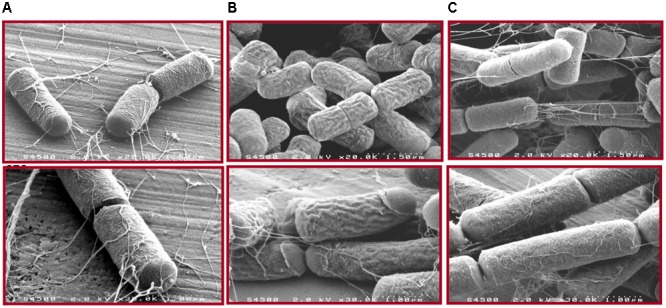
Effects of *dltX* deletion on *Bt* 407 cells. Scanning electron micrographs show exponential growth phase cells of WT **(A)**, *ΔdltX* **(B)**, and *ΔdltX* complemented cells **(C)** in Y buffer (pH 5.6). Magnifications are x20k and x30k. The dotted lines on pictures in the top panel represent a scale of 1.5 μm (x20K magnification) and those in the bottom panel represent a scale of 1 μm (x30K magnification).

We used SEM to analyze cells collected in mid-log phase to examine phenotypical differences between WT and *dltX* mutant strains. Deletion of *dltX* strongly affected cell surface morphology (**Figure 3**). WT cells had a typical rod shape with a regular and smooth surface (**Figure 3A**), whereas the *dltX* mutant cells had an irregular and wrinkled shape, and showed rib-like protrusions on their surface (**Figure 3B**). Complemented mutant cells (**Figure 3C**) showed an appearance similar to that of WT cells.

### The *dltX* Mutant Is Sensitive to Polymyxin B and Nisin *In Vitro*

**FIGURE 4:**
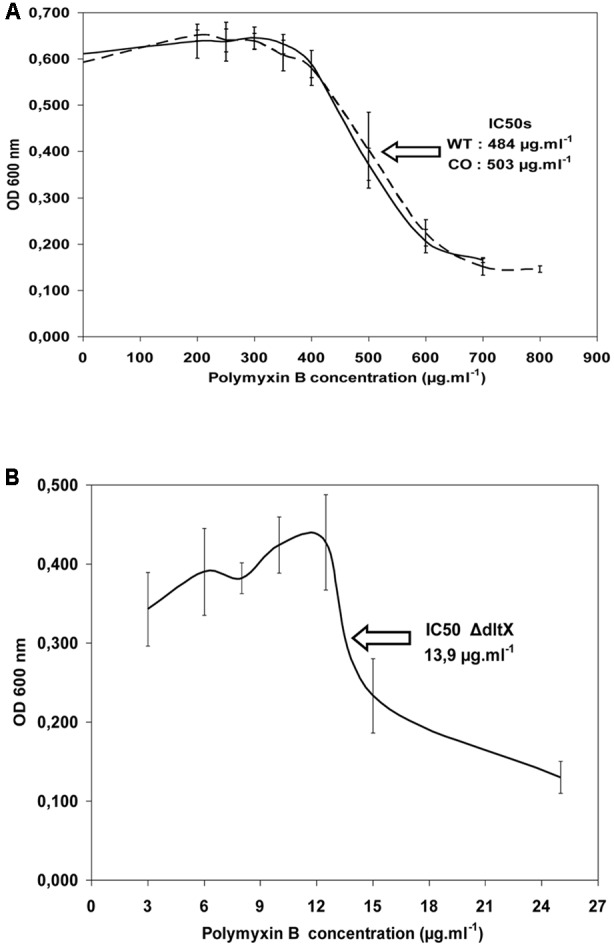
Half inhibitory concentration (IC_50_) of WT 407 (solid line) and *ΔdltX* complemented strains (CO) (broken line) **(A)**, and the *ΔdltX* mutant **(B)**. Bacterial growth was scored after 6 h of inoculation. The results shown are the means of at least three independent experiments performed in duplicate.

We compared the growth of WT *Bt* 407, *Bt* 407Δ*dltX* mutant and complemented mutant strain *Bt* 407Δ*dltX* (pHT304-18Ω*dlt)* exposed to polymyxin B or Nisin, to determine if *dltX* has a direct role in resistance to CAMPs. Addition of 150 μg polymyxin B ml^-1^ or 75 μg Nisin ml^-1^ to a growing culture caused immediate growth interruption of the mutant whereas WT and complemented strains were not affected (data not shown). Therefore, the *dltX* mutant is highly susceptible to CAMPs and its complementation with *dltX* completely restored the resistant parental phenotype. Moreover, the IC50 value of polymyxin B was 35 fold lower in the *ΔdltX* mutant than in either the WT or complemented strains. Precisely, the IC_50_ value of the *ΔdltX* mutant was 13.9 μg ml^-1^, that of the WT was 484 μg ml^-1^, and that of the complemented strain was 503 μg ml^-1^ (**Figure 4**). These results indicate that *dltX* is necessary for *B. thuringiensis* resistance to polymyxin B and that transcription of *dltABCD* alone under the control of the upstream promoter region is not sufficient to confer resistance.

### Deletion of *dltX* Leads to Attenuated Virulence in *Galleria mellonella*

**FIGURE 5:**
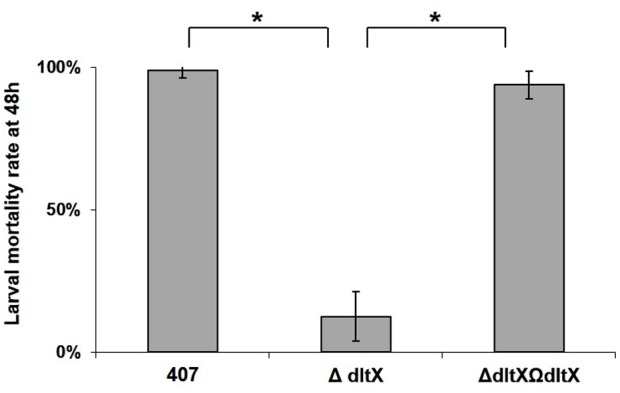
*In vivo* virulence of WT, *ΔdltX*, and complemented strains in *Galleria mellonella*. Bacterial cells were collected at OD = 1 and 1 × 10^4^ cfu were injected into the hemocoel of last instar *G. mellonella* larvae weighing about 250 mg. Infected larvae were kept at 30°C in individual Petri dishes and mortality was recorded 48 h following infection. The means and standard errors of the mean (bars) of four biological replicates are shown. Asterisks indicate a significant difference (*p* < 0.0001) determined by the χ^2^ test.

The finding that *dltX* is essential for the resistance of Bt407 to CAMPs led us to investigate the effect of its deletion on bacterial virulence in two insect models. We injected 10^4^ vegetative cells of the WT, mutant, or complemented strains into fifth instar *G. mellonella* larvae and compared their virulence by monitoring the mortality level of *Galleria* infected with the different strains (**Figure 5**). The WT and complemented strains were significantly more virulent in insects (*p* < 0.0001) than the *dltX* mutant strain (**Figure 5**). In fact, virulence in *G. mellonella* was almost completely abolished in the *dltX* mutant (13% mortality) whereas WT and complemented strains were highly virulent and gave almost 100% mortality 48 h post-infection.

### The Low Virulence of the *dltX* Mutant Is Directly Correlated with Its Sensitivity to the Humoral CAMP Response *In Vivo*

**FIGURE 6:**
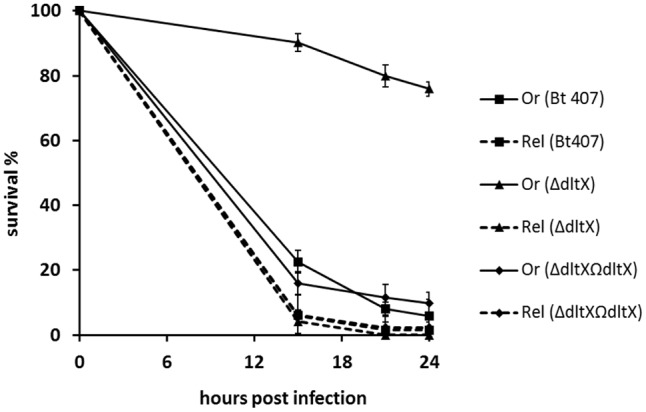
Survival analysis of *Drosophila melanogaster* adult flies infected with WT, *ΔdltX*, and complemented bacterial strains. R*elish* (Rel) flies were used as immunocompromised mutants of the Imd pathway and wild type (WT) Oregon (Or) flies were used as an immunocompetent control in these experiments. These flies were infected with WT, *ΔdltX*, or complemented strains and survival was recorded at various time points post-infection. The means and standard errors of the mean (bars) of three independent experiments are shown.

The immune system in insects comprises cellular and humoral responses that have been largely investigated in the model organism *Drosophila melanogaster* (Lemaitre and Hoffmann, 2007). The humoral response involves the infection-induced activation of NF-κB transcription factors, Dif and Relish, which in turn activate AMP encoding genes. Dif is downstream from the Toll pathway and Relish is downstream from the IMD pathway (Hedengren et al., 1999; Rutschmann et al., 2000). These signaling cascades are specifically elicited by microbial associated molecular patterns (MAMPs) that are detected by cognate pattern recognition receptors (PRRs), which are members of the Peptidoglycan Recognition Receptor and Glucan Binding Receptor families (Ferrandon et al., 2007). Notably, the Toll pathway is triggered upon sensing of Lysine (Lys)-type peptidoglycan, which is a common component of most Gram-positive bacteria, whereas the IMD pathway is activated by the mesodiaminopilmelic acid (DAP)-type peptidoglycan common to Gram-negative bacteria (Leulier et al., 2003; Ferrandon et al., 2007). The wall of all *B. cereus* group species also consists of a DAP-type peptidoglycan (Vollmer et al., 2008). We therefore, compared the virulence of WT, Δ*dltX*, and the complemented strain in WT *oregon*R and *relish* mutant flies to confirm that the loss of virulence phenotype of the Δ*dltX* mutant is associated with its susceptibility to CAMPs *in vivo*. *Bt*407 was resistant to the fly humoral response because WT and *relish* immunodeficient insects exhibited similar survival curves with high lethality of the infected flies at 15 h post-infection (**Figure 6**). Similar to findings obtained with *Galleria* larvae, survival curves of WT flies infected with the Δ*dltX* mutant showed that loss of *dltX* is sufficient to significantly impair virulence. Indeed, 75% of the flies infected with the Δ*dltX* mutant survived 24 h post-infection. Interestingly however, the virulence of the Δ*dltX* mutant was fully restored in *relish* mutant flies which do not produce CAMPs in response to the infection. This phenotype is associated with the loss of DltX because the complemented strain was as virulent as the WT strain in both WT *oregon R* and *relish* mutant flies. These findings clearly demonstrate that DltX is required for the resistance of *Bt* 407 to the humoral antimicrobial response of *Drosophila* infected by injection into the hemocoel.

### DltX Is Involved in the D-Alanylation of TAs

**FIGURE 7:**
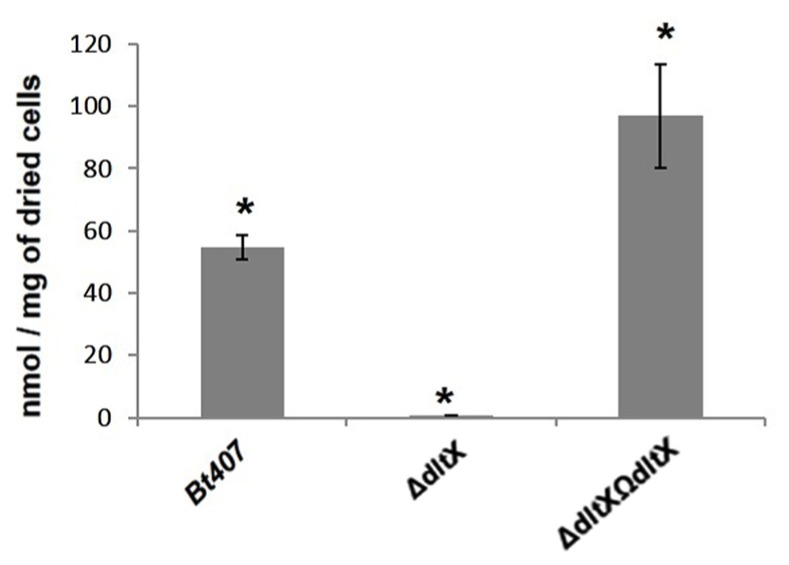
Amounts of D-alanine released from whole cells by alkaline hydrolysis for WT, mutant, and complemented strains. The mean values and standard deviations (black bars) of results from three independent experiments are shown. Asterisks indicate a significant difference (*p* < 0.0001) determined by the χ^2^ test.

We sought to determine whether the sensitivity of the *dltX* mutant to CAMPs was due to impairment in the incorporation of D-alanyl esters into TAs of the cell wall. D-Alanine was released by mild alkaline hydrolysis from whole heat-inactivated bacterial cells and quantified by HPLC analysis. Almost no D-alanine was released from the cell walls of the Δ*dltX* mutant, contrary to what is observed for the WT *Bt* 407, indicating that D-alanylation of TA was significantly impaired in the mutant. D-alanine amount released from the complemented strain was significantly higher than the one released from WT strain. This result can be explained by the copy number of the plasmid used for complementation (**Figure 7**).

### Deletion of *dltX* Significantly Alters Ionic Surface Charge

**FIGURE 8:**
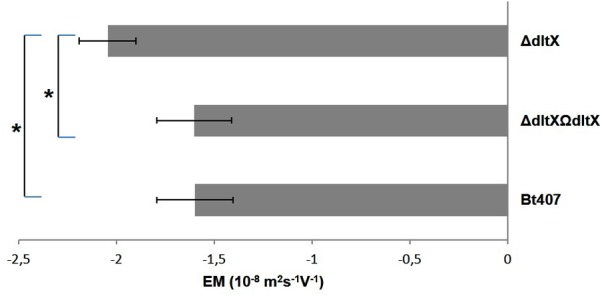
Electrophoretic mobility EM (10^-8^ m^2^s^-1^V^-1^) of WT, mutant, and complemented strains. Bacteria were suspended in 1.5 × 10^-3^ M sodium chloride and the values shown correspond to the results obtained at pH7. The mean values and standard deviations (bars) were calculated from two independent experiments with technical duplicates for each. Asterisks indicate a significant difference (*p* = 0.0007) determined by the χ^2^ test.

The loss of D-alanylation in the *dltX* mutant may result in a net change in electrical charge of the bacterial surface, which may explain the higher sensitivity of the *dltX* mutant to CAMPs both *in vitro* and *in vivo.* We measured the electrophoretic mobility (EM) of each strain to see if the deletion of *dltX* was associated with a change of the global charge at the bacterial surface. EM was significantly higher in the Δ*dltX* mutant than in the WT strain (*p* = 0.0007) (**Figure 8**), showing that deletion of *dltX* alters electrical surface charge. Thus, the Δ*dltX* strain has a high negative charge at its surface.

## Discussion

The *dlt* operon encodes proteins that are responsible for the incorporation of D-alanine into TAs, which is an important process for bacterial resistance to CAMP’s. However, the functions of several *dlt* genes remain unclear and are still under investigation. Moreover, few studies have indicated that *dltX* may be part of the *dlt* operon despite the fact that the gene is present in the genomes of 807 bacteria belonging to the Firmicutes phylum, as revealed by our *in silico* analysis. This is probably due to the fact, that, in the original genomes annotations, the small ORF preceding the *dltABCD* genes has been frequently missed due to its small size. Therefore, the involvement of the gene product of *dltX* in the function of this operon has never been investigated until now despite the fact that its sequence is highly conserved among Gram positive Firmicute family bacteria, which suggests that the product of this gene could be important and involved in the D-alanylation process.

In the present study, we used the human opportunistic and entomopathogenic bacterium *B. thuringiensis* crystal minus strain 407 and investigated the role of *dltX* in the resistance of this pathogen to cationic antimicrobial host components. In strain *Bt* 407 *dltX* is transcribed along with the other genes of the operon such as already found in several other species such as *S. aureus* or *Lactobacillus plantarum* (Koprivnjak et al., 2006; Palumbo et al., 2006; Bron et al., 2012). We first constructed a *dltX* mutant by allelic exchange, in which the *dltABCD* genes were intact and expressed under the control of their endogenous promoter. The resulting *dltX* mutant had a pleiotropic phenotype, including changes in bacterial cell morphology, high susceptibility to bacterial and insect cationic AMPs, and attenuated virulence in two insect infection models. These observations demonstrate that functional DltX is essential for resistance to CAMPs. Moreover, we found that no D-alanine was released from the cell walls of the Δ*dltX* mutant, unlike the WT or complemented strains, suggesting that the loss of *dltX* function results in the complete absence of D-alanylation. This was an unexpected finding because previous reports have claimed that the DltA-D proteins are necessary and sufficient for D-Alanine incorporation and these proteins were not inactivated in our mutant (Perego et al., 1995; Neuhaus et al., 1996; Neuhaus and Baddiley, 2003). Indeed, we have demonstrated, both by RT-qPCR and by activity of a *gfp* reporter construct, that transcription of the other genes of the *dlt* operon is not affected by the absence of *dltX.* Moreover, complementation of the mutant strain with *dltX* resulted in the complete restoration of all analyzed phenotypes. These findings suggest that DltX does not act as a *cis* or *trans* element that regulates transcription of the operon, but plays a direct biosynthetic, transport or addresser role in D-alanylation. We have also performed a complementation of the *ΔdltX* mutant with a mutated form of *dltX* in which the start codon (ATG) was replaced by a stop codon (TGA) thus impeding the translation of the putative DltX protein (data not shown). Unlike the native *dltX* sequence, this mutated form of *dltX* could not restore the parental phenotype of resistance to CAMPs and virulence toward *G. mellonella*. However, the exact function of DltX remains to be elucidated.

We also observed that disruption of *dltX* strongly affected the cell morphology of *B. thuringiensis* since the Δ*dltX* mutant strain presented irregular shapes which were not observed in the parental strain (**Figure 3**). Such dramatic changes in cell morphology have not been reported for other Δ*dlt* mutants of gram positive bacteria, except for *L. plantarum.* Indeed, scanning and transmission electron microscopy (SEM and TEM) of a *L. plantarum* Δ*dlt* mutant showed perforations of the cell envelope (Palumbo et al., 2006). The authors suggested that this effect may result from a high rate of autolysis. We observed no differences between the growth curve of the WT parental strain and that of the Δ*dltX* mutant (data not shown). This indicates that the surface modifications are unlikely to influence the rate of autolysis, at least *in vitro* and under the conditions tested. Moreover, we showed that the cell surface of the Δ*dltX* mutant was significantly more negatively charged than that of the WT strain. This is not surprising because the cell wall is predicted to be largely more anionic in the absence of positively charged D-alanyl esters. However, in contrast with our results, Giaouris et al. (2008) found that the level of D-alanylation in the cell wall of *Lactococcus lactis* did not significantly modify the global surface charge. They suggested that most D-alanyl substituents of TAs are located inside the cell wall and are not exposed at the cell surface, which may explain why electrophoretic mobility was not affected in this mutant strain. Thus, the inactivation of the *dlt* operon has a wide range of physiological consequences in different bacteria, but the absence of D-alanyl esters in the TAs does not result in a clear morphological phenotype that is common to all the bacterial species harboring a *dlt* operon except their higher sensitivity to CAMPs (Peschel et al., 2000; Fabretti et al., 2006).

*DltA, dltB, dltC, or dltD* mutants of several bacteria show high susceptibility to CAMPs and are killed by peptides of host defense mechanisms (Poyart et al., 2003; Kovács et al., 2006; Abi Khattar et al., 2009; McBride and Sonenshein, 2011). We now show that DltX is also involved in the response to antimicrobial peptides. Indeed, inactivation of *dltX* alone (without affecting the expression of *dltABCD*) also substantially impaired the resistance of *B. thuringiensis* to CAMPs (**Figure 4**) and significantly attenuated *B. thuringiensis* virulence in insect larvae (**Figures 5, 6**).

We also took advantage of the *Drosophila melanogaster* infection model to demonstrate the prominent role of the D-alanylation of TAs during bacterial resistance to the antimicrobial response *in vivo*. Indeed, both *Oregon R* WT, and *relish* mutant flies (that do not produce AMPs in response to infection), were highly susceptible to infection by WT *Bt* 407. By contrast, virulence of Bt 407Δ*dltX* was significantly attenuated, as revealed by the survival of a large proportion of *oregon R* WT adult flies infected with the Δ*dltX* mutant, but retained its pathogenic effect on *relish* mutants (**Figure 6**). Hence, the process of D-alanylation may be essential for the persistence, development, and multiplication of the bacteria in an antimicrobial hostile environment. These findings also indicate that in the absence of antimicrobial peptides, the Δ*dltX* mutant possesses efficient resistance strategies to deal with the other mechanisms of host defense, such as cellular or melanization responses (Nielsen-LeRoux et al., 2012). It will be interesting to investigate these strategies in the *B. cereus–Drosophila* interaction model.

**FIGURE 9:**
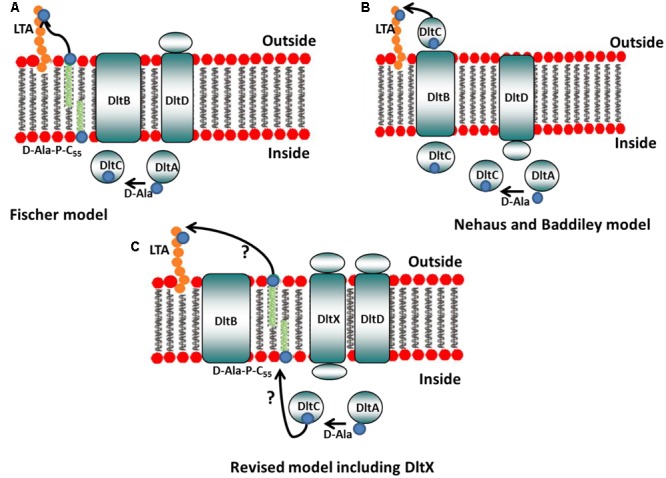
Models of D-alanine substitution of LTA. **(A)** Fischer model: DltA ligates D-alanine (small blue circle) onto the carrier protein DltC. DltB then transfers D-alanine from DltC to undecaprenyl-phosphate (C_55_-P) to produce D-Ala-P-C_55._ This lipid linked intermediate is, then, flipped accross the membrane, whereas DltD, active at the outer side of the membrane, transfers D-Ala to LTA (adapted from reference 5). **(B)** Neuhaus and Baddiley model. DltA ligates D-alanine (small blue circle) onto the carrier protein DltC. DltD is thought to facilitate D-alanine ligation to DltC and DltB is believed to be involved in the translocation of Alanylated-DltC across the membrane where it may then transfer D-alanine directly onto LTA (adapted from reference 5). **(C)** Revised model including DltX: our results show that DltX is essential for D-alanylation of TAs. Therefore, DltX was included onto the existing models to obtain a more complete picture of the mechanism of D-alanine incorporation into the cell wall polymers of Gram-positive bacteria. DltX, due to its alpha-helical structure prediction and the presence of both extracellular and cytosolic domains, can theoretically interact with the four other proteins of the operon (see hypotheses in the text). However, the exact functions of DltX as well as DltB and DltD in this process remain to be elucidated.

Moreover, based on our results, we propose that the two existing models that describe the functions of the proteins encoded by *dltABCD* should be amended to include *dltX*, to obtain a complete picture of the mechanism of D-alanine incorporation into the cell wall polymers of Gram-positive bacteria (**Figure 9**). This model takes into account the recent findings of Reichmann et al. (2013) that showed that DltC does not cross the membrane and that DltD is anchored to the outside of the cell. Therefore, it is unlikely that the carrier protein DltC is involved in the final step of D-alanylation on the outside of the cell, and that DltD, which is active at the outer side of the membrane, facilitates D-alanine ligation to DltC, as proposed by Neuhaus and Baddiley. DltB is predicted to be an integral membrane protein with 12 transmembrane spanning alpha helixes. In this model, DltX, due to its alpha-helical structure prediction and the presence of both extracellular and intracytoplasmic domains, can theoretically interact with any of the four other proteins of the operon (DltA, DltB, DltC, or DltD), or even with several of them. We can consider first, that it is the intracytoplasmic domain of DltX which is involved, and which interact with one of the intracellular components (DltA or DltC). Could DltX play the role of the intermediate undecaprenol-phosphate (which has not yet been confirmed experimentally) or could it help in the transfer of D-alanine from DltC to this lipid linked intermediate? Another possibility is that DltX plays the role of a flippase which flips the D-alanylated undecaprenol-phosphate across the membrane. An alternative, is that the transmembrane segment of DltX interacts with one of the transmembrane domains of DltB and contributes to the transfer of molecules (such as D-alanine) through the membrane channel formed by DltB. A fourth possibility is that it is the extracellular domain which is involved in the activity of DltX. This domain could interact with the D-alanylated undecaprenol-phosphate, once it is exposed on the outside of the membrane, and be necessary, together with DltD, for the transfer and/or ligation of D-alanine to TAs. It is interesting to note that only two amino acids are completely conserved in all DltX proteins described to date, one is located in the transmembrane alpha helix and the other in the extracytoplasmic domain. It is important now to confirm the interaction of DltX with one of these proteins. Moreover, our results show that the wall structure is strongly affected in the *dltX* mutant; this might indicate that DltX could interacts with other proteins or enzymes independent of the *dlt* operon, involved in the synthesis of some elements of the cell wall.

## Conclusion

Our data clearly demonstrate that the synthesis of D-alanyl-TAs cannot be accomplished only by the concerted action of the four proteins encoded by the *dltA, dltB, dltC*, and *dltD* genes, as it frequently suggested in many studies. We have shown that the gene product of *dltX* is also essential in this process. Future studies addressing the mechanism of D-alanine incorporation into TAs should take into account that DltX is also necessary and essential for the the D-alanylation of TAs. However, additional work is now needed to elucidate the function of DltX and with which other(s) protein(s) of the operon it interacts.

## Author Contributions

RK: designed experiments, performed the experiments, analyzed and wrote the manuscript, AR: performed the experiments, IJ: performed the experiments, ZA: performed the experiments, PC and M-PC-C: conceived experiments and analyzed data, CN-L and DL: conceived experiments and analyzed data, LC: designed experiments and analyzed the data, MK and VS-B: conceived and designed the study, analyzed the data and wrote the manuscript.

## Conflict of Interest Statement

The authors declare that the research was conducted in the absence of any commercial or financial relationships that could be construed as a potential conflict of interest.
